# Delayed Emergence of Isolated Secondary Adrenal Insufficiency: A Case Report

**DOI:** 10.7759/cureus.71209

**Published:** 2024-10-10

**Authors:** Alexa Ovalles Lacruz, Natalie Mesa, Steven T Vassil, Angela Blanco Guertin, Deepa Sharma

**Affiliations:** 1 Medical Education, Florida International University, Herbert Wertheim College of Medicine, Miami, USA; 2 Family Medicine, Baptist Health of South Florida, Miami, USA

**Keywords:** corticosteroid replacement therapy, hydrocortisone, hyponatremia, isolated adrenal insufficiency, isolated adrenocorticotropic hormone deficiency

## Abstract

Secondary adrenal insufficiency (SAI) is a rare condition caused by adrenocorticotropic hormone (ACTH) deficiency, which disrupts ACTH secretion by the pituitary gland and can lead to chronic hyponatremia. However, idiopathic delayed onset of isolated adrenal insufficiency without triggering factors is an unusual finding and poses a challenging diagnosis. We present the case of an 80-year-old woman with hypothyroidism, hypertension, previous tobacco use, and squamous cell carcinoma of the ankle who presented with weakness and fatigue. Blood work showed hyponatremia (126 mmol/L), nonfasting blood glucose of 139 mg/dL, and elevated erythrocyte sedimentation rate (ESR, 83 mm/hr). Initial treatment included urea administration and water restriction. Initial imagining included a CT of the chest which revealed mediastinal lymphadenopathy, leading to suspicion of the syndrome of inappropriate antidiuretic hormone (SIADH) from malignancy. Despite increasing urea and adding NaHCO_3_, sodium levels dropped further. Tolvaptan was administered but showed no improvement. Endocrinology recommended hydrocortisone sodium succinate (Solu-Cortef) leading to sodium normalization and symptom resolution. A full pituitary workup revealed low dehydroepiandrosterone sulfate (DHEA-S), and abdominal CT showed atrophic adrenal glands. MRI of the sella turcica was normal with no evidence of a mass. The patient was discharged on an oral course of Solu-Cortef with stable sodium levels (139 mEq/L). This is among the first reported cases of unprovoked isolated adrenal insufficiency with abnormal cortisol, ACTH, DHEA-S levels, and normal renin and aldosterone levels. Diagnosis is often delayed due to nonspecific symptoms. Once identified, corticosteroid replacement therapy is the gold standard for management.

## Introduction

Secondary adrenal insufficiency (SAI) is a rare, potentially life-threatening condition resulting from a deficiency in adrenocorticotropic hormone (ACTH) produced by the anterior pituitary. The typical presentation includes chronic euvolemic hyponatremia, postural hypotension, lethargy, depression, anorexia, and weight loss. The ACTH deficiency may be isolated or occur in conjunction with other pituitary hormone deficiencies. SAI characterized by a low ACTH in the absence of structural deficits in the pituitary is referred to as isolated adrenal insufficiency. Some cases are thought to result from an underlying autoimmune process or undiagnosed genetic cause. 

Previous oncological case reports have shown an acute onset of SAI potentially resulting from immune-related adverse events triggered by immune checkpoint inhibitors [1-5]. In other instances, individuals undergoing long-term hemodialysis have developed ACTH deficiency [6]. However, idiopathic delayed onset of isolated adrenal insufficiency in the absence of triggering factors has not been reported. These cases often present with nonspecific manifestations, complicating clinical detection, diagnosis, and management. This underscores the need for heightened clinical awareness and thorough diagnostic evaluations of hyponatremic cases to ensure timely and effective treatment. Although the etiology of SAI may be difficult to assess due to its varied clinical presentations, swift treatment with glucocorticoid replacement therapy is crucial to avoid long-term neurologic sequelae of hyponatremia. Our case report highlights the successful management strategy employed in addressing ACTH deficiency-induced euvolemic hyponatremia, highlighting the pivotal role of timely diagnosis and targeted intervention in improving patient outcomes.

## Case presentation

This is the case of an 80-year-old female with a past medical history of hypothyroidism, hypertension, esophageal spasms, peripheral artery disease, neuropathy, previous tobacco use, and squamous cell carcinoma of the left ankle status post resection one year prior who presented to the emergency department complaining of generalized weakness and fatigue. In the emergency department, the patient was admitted due to altered mental status and a notable ulcer to her left foot and ankle accompanied by purulent drainage. On initial presentation, she received 1 L of normal saline fluid bolus for resuscitation and ceftriaxone and vancomycin for possible cellulitis. Her vital signs were blood pressure (BP), 141/75; heart rate (HR), 84/min; respiratory rate (RR), 18/min; and temperature (T), 37.9°C. She reported a one-week history of nausea, vomiting, and febrile episodes. On initial evaluation, the patient exhibited hyponatremia (sodium: 126 mEq/L) and mildly elevated inflammatory markers (erythrocyte sedimentation rate (ESR), 83 mm/hr) but no leukocytosis (white blood cells, 6.93 K/uL). The other laboratory results including blood urea nitrogen (BUN), 12 mg/dL; creatinine, 0.56 mg/dL; and potassium, 4.1 mmol/L depicted normal kidney and renal function. Hemoglobin was 11.3 g/dL, platelets were 163 K/uL, and lactic acid was 1.1 mmol/L.

The patient presented with lethargy and declining functional status, highly suspicious of underlying metabolic encephalopathy in the setting of electrolyte imbalance or as a side effect of gabapentin for neuropathy involving both lower extremities. Nephrology was consulted and suggested an initial diagnosis of hypotonic hyponatremia of unclear etiology likely secondary to intravascular volume depletion due to the initial administration of the IV fluid bolus given. She was initially managed with an oral dose of urea 15 g twice daily and placed on a free water restriction of 800 cc/day without improvement in sodium levels. According to the nursing staff, the patient had an episode of choking and turned "purple in color." Therefore, a CT of the chest was requested to rule out pulmonary embolism. Imaging reported clear lung fields with bibasilar airspace opacities probable of atelectatic changes along with mediastinal lymphadenopathy. The subsequent recommendation was to consider the syndrome of inappropriate antidiuretic hormone (SIADH) as the etiology of her hyponatremia in the setting of unknown lung malignancy considering her former smoker status, with a five-pack-year history although having stopped smoking approximately 20 years ago.

On the second day of admission, the patient had worsening hyponatremia (at 124 mEq/L) and anemia (Hb, 10.6 g/dL) despite increasing urea 15 g to three times daily and adding sodium bicarbonate 1,300 mg twice a day. On day three of admission, due to poor response to previous pharmacotherapy, the decision was made to stop urea and begin a trial of a low-dose vasopressin antagonist, tolvaptan (7.5 mg). Further evaluation was performed for suspected destruction or dysfunction of the adrenal cortex. Initial serum AM cortisol level was 1.2 mcg/dL (normal at 08:00, 5.0-22.4 mcg/dL). However, the provisional diagnosis of adrenal insufficiency was of unclear etiology considering the patient was fatigued and lethargic but hemodynamically stable-BP 121/86 mmHg-not hypotensive, with normal glucose levels (109 mg/dL) and no other clinical manifestations of hypopituitarism. Despite treatment, her mental status continued to worsen with increased lethargy and inability to answer questions or follow commands during physical examination. Neurological examination revealed normal cranial nerves. An arterial blood gas (ABG) sample was taken, and it revealed patient was clinically in nonanion gap metabolic acidosis with a negative base excess (Table 1). As a result of her decreased pO_2_ on ABG, the patient was placed on supplemental 4 L nasal cannula for one day with appropriate saturation (>98%).

**Table 1:** Arterial blood gases Ca: Calcium ion; Cl: chloride ion; HCO_3_: bicarbonate; K: potassium ion; Na: sodium ion; pCO_2_: partial pressure of carbon dioxide; pO_2_: partial pressure of oxygen; sO_2_: oxygen saturation

Blood gas values	Value	Normal range
pH	7.46	7.35-7.45
pCO_2_, mm Hg	28	35-45
pO_2_, mmol/L	58.2	83-108
Na, mmol/L	123.4	136-146
K, mmol/L	3.98	3.4–4.5
Ca, mmol/L	1.16	1.15-1.29
Cl, mmol/L	94	98-106
HCO_3_, mmol/L	19.4	22-26
sO_2_, %	92.1	95-99
Base excess, mmol/L	-3.2	-2 to +2

On the fourth day of admission, repeat low AM total cortisol was 0.8 mcg/dL. Sodium levels continued at 126 mEq/L. Due to unresponsiveness after two courses of tolvaptan, endocrinology recommended starting corticosteroid replacement therapy with hydrocortisone sodium succinate (Solu-Cortef) 25 mg IV every eight hours. Accordingly, further anterior pituitary hormone and adrenal axis assessment was performed (Table 2).

**Table 2:** Hormonal panel ACTH: adrenocorticotropic hormone; DHEA-S: dehydroepiandrosterone sulfate; FSH: follicle-stimulating hormone; FT3: free triiodothyronine; FT4: free thyroxine; GH: growth hormone; LH: luteinizing hormone; PRL: prolactin; TSH: thyroid-stimulating hormone

Hormone tested with unit	Result	Reference range
LH, mIU/mL	12.7	Females (follicular phase): 1.9-12.5
		Females (midcycle peak): 8.7-76. 3
		Females (luteal phase): 0.5-16.9
		Females (pregnant): <0.1-1.5
		Females (post-menopausal): 15.8-54.0
		Females (contraceptive): 0.7-5.6
FSH, mIU/mL	50.2	Females (follicular phase): 2.5 -10.2
		Females (midcycle peak): 3.4-33.4
		Females (luteal phase): 1.5-9.1
		Females (pregnant): <0.3
		Females (postmenopausal): 23.0-116.3
		Males (13-70 years of age) 1.4-18.1
TSH, μIU/L	3.93	0.4-4.0
FT4, ng/dL	1.7	0.9-1.7
PRL, ng/mL	8.4	Females, non-pregnant: 2.8-29.2
		Females, pregnant: 9.7-208.5
		Females, menopausal: 1.8-20. 3
		Males: 2.1-17.7
DHEA-S μg/dL	9.9	Levels vary by age and sex: female ranges
		Ages 18-29: 45-320
		Ages 30-39: 40-325
		Ages 40-49: 25-220
		Ages 50-59: 15-170
		Ages 60-69: 13-130
		Ages 70 and older: 17-90
ACTH, pg/mL	3.0	8:00 am: varies from 10 and 65
Cortisol, μg/dL	0.8	Specimen collected at
		08:00, 5.0-22.4
		16:00, 3.0-16.0
		20:00, <50% of the 08:00 values
Renin, ng/mL/hr	0.820	0.167-5.380
Aldosterone, nd/dL	< 1	0.0-30.0

On day five of hospitalization, mental status improved significantly with the latest sodium at 131 mEq/L. Laboratory results revealed an inappropriately low ACTH of 3.0. pg/mL. The remaining pituitary hormone axis revealed FSH, LH, and prolactin levels within normal limits (Table 2). Thyroid peroxidase antibodies (TPA < 28.0 U/mL: normal) were negative. For the adrenal axis evaluation, serum adrenal hormone levels revealed decreased DHEA-S of 9.9 ug/dL with normal renin and aldosterone values (Table 2). In the setting of isolated ACTH pituitary dysfunction, the plan was to continue intravenous (IV) hydrocortisone for 72 hours before switching to oral hydrocortisone. On day six, oral hydrocortisone was decreased to 25 mg every 12 hours after optimal response, and sodium levels consistently trended upward (Na, 138 mEq/L). The patient denied any prior use of oral or injectable steroids. Etiology remained unclear. 

**Figure 1:**
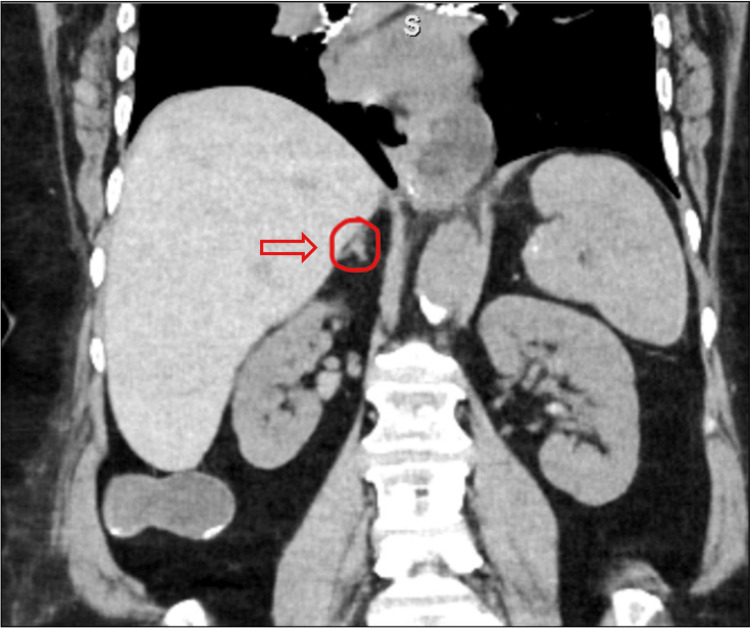
Abdominal CT showing atrophy of adrenal glands

**Figure 2:**
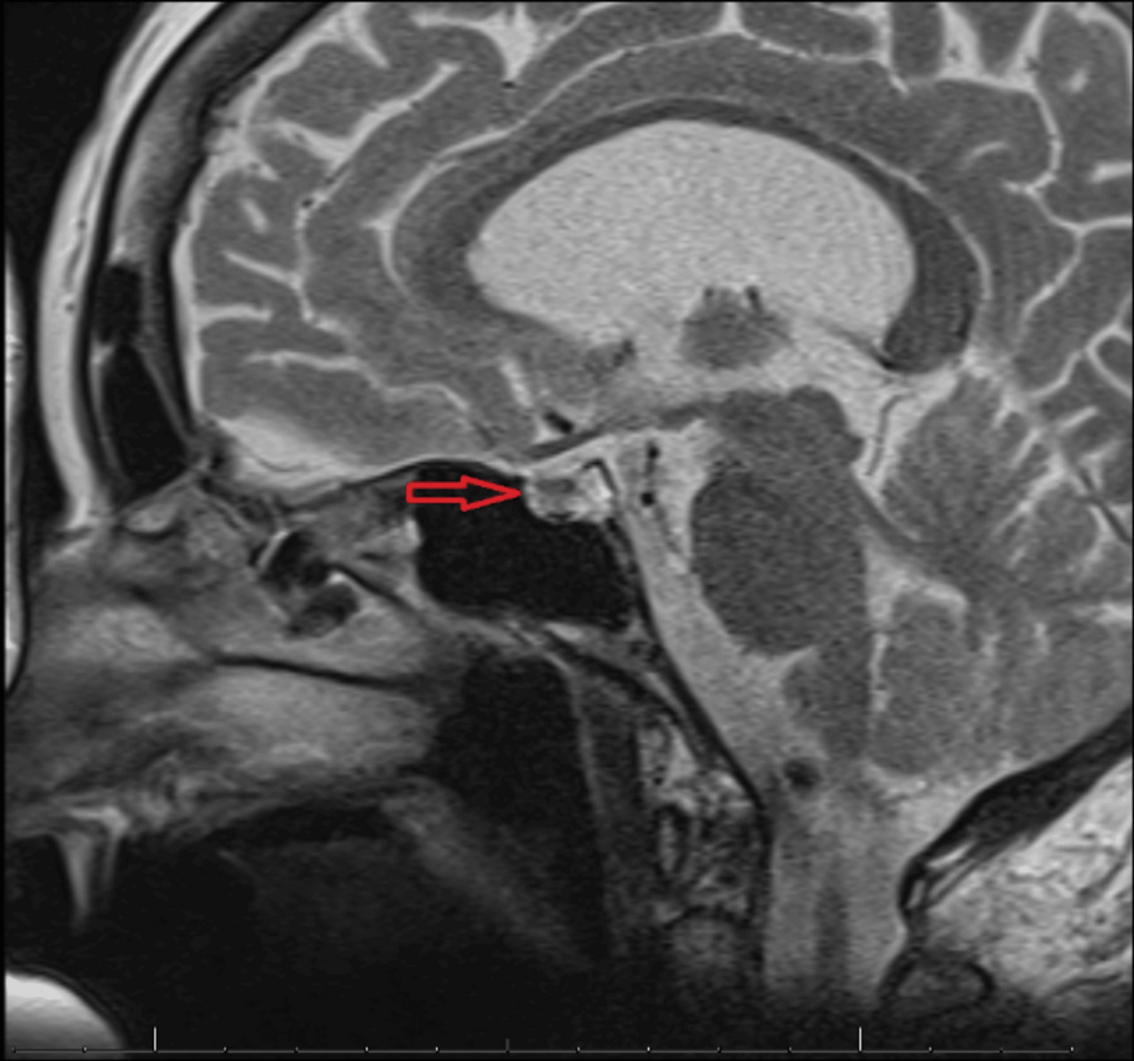
Normal noncontrast MRI of the sella

On further follow-up, an abdominal CT was requested and revealed an atrophic appearance of the bilateral adrenal glands, which may be seen in the setting of adrenal insufficiency (Figure 1). An initial brain MRI previously ordered at the time of admission, revealed a 4 mm focus of hypoenhancement within the right lateral aspect of the adenohypophysis. Follow-up MRI without contrast focused on the pituitary revealed no mass effect, no small lesion, and no signs of bleeding; along with a normal sella turcica (Figure 2). On the seventh day of admission, IV hydrocortisone was switched to an initial regimen of oral hydrocortisone 10 mg in the morning and 5 mg in the afternoon and was later increased to 15 mg AM and 10 mg PM. The final optimal dose of glucocorticoid replacement at the time of discharge (day 10) was twice a day with hydrocortisone sodium succinate (Solu-Cortef) 50 mg by mouth in the morning followed by one course of Solu-Cortef 10 mg by mouth in the afternoon between 2 and 3 PM. The underlying etiology of her acute presentation remains unclear.

No other known triggers explain the delayed emergence of her isolated adrenal insufficiency. Her sodium level on discharge was 139 mEq/L. The patient was discharged with strict guidelines to reduce the risk of adrenal crisis during times of illness, injury, or stress and recommended lifetime avoidance of thiazide, angiotensin-converting enzyme inhibitors, proton pump inhibitors, nonsteroidal anti-inflammatory drugs, and selective serotonin inhibitors. No stimulation tests were performed during this hospitalization. She was instructed to follow-up on an outpatient basis for stimulation testing, anti-pituitary antibodies, and further workup of other autoimmune diseases.

## Discussion

The patient reported here illustrates an uncommonly seen case of unprovoked Isolated ACTH deficiency. Her presenting clinical symptoms of lethargy, confusion, and generalized weakness initially diagnosed as metabolic encephalopathy in the setting of significant hyponatremia did not improve after a trial of fluid restriction, urea, and tolvaptan therapy. During subsequent workup, the patient was diagnosed with SAI without known trigger factors. Corticosteroid replacement therapy was effective for the management of her acute electrolyte imbalance. 

SAI is an uncommon and potentially fatal disorder caused by tumors, head injuries, or genetic defects that disrupt the pituitary gland’s normal hormonal secretion patterns. These causes can hinder the appropriate secretion of corticotropin (ACTH) [7]. The prevalence of SAI is estimated to range between 150 and 280 cases per million worldwide, with corticosteroid-induced insufficiency being the most common etiology [8]. The dysfunction of ACTH secretion can occur on its own or alongside other pituitary hormone deficiencies, leading to hypopituitarism [7]. Rare loss-of-function mutations within the POMC gene have been shown to cause SAI, playing a key role in metabolic complications [9]. Our patient's case is noteworthy for its lack of a clear etiology consistent with documented causes of SAI and isolated ACTH deficiency. Genetic investigation may provide insights, although specific mutations in the POMC gene have not been identified in this case.

Isolated ACTH deficiency represents a rare endocrine disorder characterized by impaired secretion of ACTH from the anterior pituitary, ultimately resulting in decreased cortisol levels in the body. Studies have revealed that patients with cortisol deficiency have an increased mortality rate secondary to metabolic, cardiovascular, infective, and neoplastic conditions [10]. Our patient experienced a rapid decline in mental status and developed hyponatremic encephalopathy, underscoring the severe consequences of cortisol deficiency. The clinical manifestation of SAI lacks specificity and often causes delayed diagnosis. Symptoms include fatigue, decreased appetite, hypoglycemia, hyponatremia, muscle weakness, and loss of consciousness. Patients with SAI do not have dehydration, and hypotension is less prominent, as it was in our case [7].

Initially, there was a high suspicion of SIADH-induced hyponatremia considering her history of hypothyroidism, tobacco use, and incidental mediastinal lymphadenopathy on the CT scan of the chest. Although urea is not widely used worldwide for the treatment of SIADH, a systematic review suggested that urea might be an effective and inexpensive treatment option as recommended previously by the 2014 joint guidelines on diagnosis and treatment of hyponatremia by the European Renal Association [11]. Our patient did not respond to either urea or a trial of selective arginine vasopressin (AVP) V2 receptor blocker-tolvaptan. Failure to induce free-water diuresis prompted evaluation of other etiologies.

In the absence of risk factors, glucocorticoid deficiency was suspected as the cause of our patient’s hypotonic hyponatremia. Workup for the destruction or dysfunction of the adrenal cortex workup includes measuring AM cortisol, with values <3 mcg/dL highly suggestive of adrenal insufficiency. Further, the measurement of plasma ACTH is the mandatory step in the differential diagnosis of adrenal insufficiency [12]. In our case, no deficiency in the secretion of other anterior pituitary hormones was observed, and with isolated low ACTH, the diagnosis of SAI was investigated. The insulin tolerance test (ITT) is the gold standard for the diagnosis of SAI but is not widely used because the testing procedure is burdensome, expensive, and carries safety risks [13]. In our case, pituitary imaging was the first point of call and an acceptable alternative to the traditional metyrapone stimulation test [14]. Initial brain MRI revealed a 4 mm focus of hypoenhancement in the adenohypophysis. However, pituitary incidentalomas are frequently encountered and are unlikely to cause SAI unless >1 cm. No other abnormalities were noted, similar to several cases confirmed to have isolated ACTH deficiency [15]. The absence of abnormalities on imaging does not rule out an autoimmune cause and may suggest an early stage of the disease where pituitary changes are not yet detectable [15]. 

Management is challenging and depends on the patient’s severity of acute presentation. Once the diagnosis is obtained, it is critical to begin management with the administration of physiologic doses of hydrocortisone in the quantity and timing that mimic the normal patterns of cortisol secretion (about 2-3 times per day). The 2016 Endocrine Society clinical guidelines [16] recommend hydrocortisone as the glucocorticoid replacement therapy. Hydrocortisone is the preferred treatment of choice due to its short-acting mechanism and optimal dose titration in small increments (2.5 to 5 mg). Bancos et al. state that there is no reliable biochemical marker to determine the correct glucocorticoid replacement dose. Consequently, adjustments are made based on clinical judgment, subjective assessment of symptoms, and signs of either under- or over-replacement of glucocorticoids [12]. Our patient responded to an initial regimen of hydrocortisone 25 mg IV every eight hours leading to sodium normalization and symptom resolution. After several dose adjustments during hospitalization, the optimal dosing at discharge was oral hydrocortisone sodium succinate (Solu-Cortef) 50 mg in the morning and 10 mg in the afternoon (between 2 and 3 pm).

Preventing and treating adrenal crises is of utmost importance to avoid fatal outcomes. Due to the rarity of adrenal insufficiency, many physicians are not familiar with its management, often resulting in critical delays in emergency treatment. Patients and relatives should be educated on the correct adjustment of glucocorticoid replacement (stress dosing/sick day rules), symptom awareness, and the use of steroid emergency cards and medical alert bracelets.

## Conclusions

Herein, this case exhibits the complex management of late-onset isolated ACTH deficiency presenting with hyponatremia and metabolic encephalopathy in an 80-year-old female without clear etiology or recognized trigger. The absence of identifiable triggers, such as tumors, autoimmune diseases, or previous corticosteroid use, emphasizes the need for thorough diagnostic evaluation in patients with unexplained hyponatremia. In this case, the patient’s condition improved markedly with prompt initiation of hydrocortisone therapy, demonstrating the essential role of corticosteroid replacement in managing adrenal insufficiency. Given the rarity of this condition, our patient’s diagnosis adds to the limited literature on its occurrence. Further, it emphasizes the need to increase the level of consideration for uncommon differential diagnoses during the assessment of persistent hyponatremia with vague symptomatology. Clinicians must remain vigilant for subtle signs and perform timely hormone evaluations to initiate life-saving interventions. The need for lifelong steroid therapy and patient education on avoiding adrenal crises further highlights the long-term management challenges associated with this condition.
